# Association Between HIV Risk and Health Care Access Among U.S. Adults in the South: Insights from the 2022 Behavioral Risk Factor Surveillance System

**DOI:** 10.1007/s10461-025-04929-y

**Published:** 2025-10-28

**Authors:** Precious Patrick Edet, Azad R. Bhuiyan, Edith Ezekwe, Abdul R. Shour, Trisha Arnold, Amy Nunn

**Affiliations:** 1 William Magee Institute for Student Wellbeing, University of Mississippi, University, MS 38677 USA; 2 Department of Public Health, University of Mississippi, University, MS 38677 USA; 3 Department of Epidemiology and Biostatistics, Jackson State University, Jackson, MS 39213 USA; 4 School of Nursing, University of Mississippi Medical Center, Jackson, MS 39216 USA; 5 Department of Medicine, University of Mississippi Medical Center, Jackson, MS 39216 USA; 6 Essentia Institute of Rural Health, Duluth, MN 55805 USA; 7 Department of Psychiatry and Human Behavior, Brown University Health, Providence, RI 02912 USA; 8 Department of Behavioral and Social Health Sciences, Brown University, Providence, RI 02912 USA

## Abstract

In the United States (U.S.), the highest burden of new HIV diagnosis continues to occur in Southern states. Healthcare access among at-risk populations is crucial to mitigate HIV transmission, yet data on the association between HIV risk and healthcare access is limited. This study examined the association between HIV risk, (i.e., injecting any drug other than those prescribed, engaging in transactional sex, receiving treatment for a sexually transmitted infection, having condomless anal sex, or having four or more lifetime sexual partners—all within the past year) and healthcare access among adults in the South, adjusting for covariates. The 2022 Behavioral Risk Factor Surveillance System for Southern states was analyzed, and 191,266 respondents participated. Rao-Scott Chi-square tests and weighted logistic regression analyses using complex sampling design were performed in SAS v. 9.4. Findings suggest that U.S. adults in the South at risk for HIV had higher odds of not having health insurance coverage (OR=1.55; 95% CI: 1.34–1.79), not having a personal doctor (OR=2.00; 95% CI: 1.78–2.25), delaying routine check-ups for a year or more (OR=1.75; 95% CI: 1.57–1.96), and being unable to afford medical care in the past year (OR=2.26; 95% CI: 2.00–2.56), compared to those not at risk, for unadjusted analyses. After adjusting for covariates, findings suggest that adults in the South at risk for HIV had 1.69 higher odds of being unable to afford medical care in the past year due to financial constraints (95% CI: 1.45–1.97) compared to those not at risk. Among adults at risk for HIV in the South, enhancing access to HIV preventive services, particularly during public health emergencies, has the potential to mitigate HIV transmission risks and reduce the associated financial burden. This association warrants reevaluation in the post-pandemic era to guide future prevention efforts effectively.

## Introduction

HIV (Human Immunodeficiency Virus) poses a serious public health challenge in the United States (U.S.) with more than 700,000 lives lost to the disease since the start of the epidemic in 1981 [1]. There are approximately 1.2 million people living with HIV in the U.S., and about 13% (1 in 7) of these individuals do not know that they have HIV [2]. The South, consisting of Alabama, Arkansas, Delaware, Florida, Georgia, Kentucky, Louisiana, Maryland, Mississippi, North Carolina, Oklahoma, South Carolina, Tennessee, Texas, Virginia, West Virginia, experiences the highest HIV burden as approximately 52% of all HIV diagnosis occur in this region [3]. The Ending the HIV Epidemic in the U.S. Initiative (EHE) by The U.S. Department of Human and Health Services (DHHS) identified seven priority states in its plan to reduce new HIV infections in the United States, with six out of the seven states being from the South [1, 4].

Several populations are disproportionately affected by HIV, thereby putting them at a higher risk of HIV. Various factors including individual factors such as a person’s geographical location, subpopulation group, and behavior contribute to this increased risk [5]. For example, living in an area with a high HIV prevalence, sharing needles, engaging in transactional sex, and having multiple sexual partners, puts a person at a higher risk of contracting HIV. According to DHHS [5], populations at increased risk of HIV include people who report gay, lesbian, and bisexual orientations; men who have sex with men (MSM); transgender women; Black women, individuals aged 13–24; and people who use drugs.

Epidemiological investigation by the Centers for Disease Control and Prevention (CDC) into populations at risk for HIV revealed that Black/African American and Hispanic/Latino individuals are disproportionately affected by HIV, accounting for approximately 70% of estimated new infections in 2022 [6]. Additionally, men remain at higher risk of HIV compared to women, making up almost 80% of newly reported infections in 2022 [7]. In 2022, gay and bisexual men, along with other men who have sex with men (MSM), accounted for 67% of all new HIV infections and 83% of all people living with HIV [8]. People who inject drugs (PWID) accounted for 2,300 (7%) of new HIV infections in 2022, with men and women comprising 4% (1,300) and 3% (1,000), respectively [8].

Healthcare access is defined as the ease and opportunity for individuals to obtain and receive medical services, treatment, and healthcare resources in a timely manner [9, 10]. It represents a structural factor that may contribute to HIV risk and related health disparities, particularly in the South, where limited access to care and high poverty rates are prevalent.

Mississippi, for instance, reported an HIV incidence rate of 18 cases per 100,000 people in 2022, compared to the national rate of 13 cases per 100,000 [11]. Additionally, the state consistently ranks among states with poor healthcare access, characterized by limited health insurance coverage and shortages of healthcare providers [12, 13]. In 2018, 17% of adults in Mississippi were unable to afford necessary medical care, exceeding the national average of 13%, and in 2019, over a quarter of Mississippi adults (26%) lacked a personal healthcare provider, compared to the national average of 23%, according to Kaiser Family Foundation [12]. Similarly, in 2022, Alabama reported an HIV incidence rate of 16 cases per 100,000 people, higher than the national rate of 13 per 100,000 [14]. Despite this high rate, health care access ranks the second greatest current health concern in the state, with 18% of Alabamians being uninsured in 2019 compared to the national average of 14% [15]. Furthermore, approximately 1 in 6 (16%) women of childbearing age (18–44) in Alabama lack health insurance [16].

The CDC highlights that structural issues such as poverty and limited access to high-quality health care impact health outcomes, resulting in health inequities [8]. According to Maxwell et al. [17], lack of health insurance serves as a predictor of late HIV diagnosis, higher numbers of infected clients, and lower HIV testing rates. Poor access to HIV prevention services (e.g., pre-exposure prophylaxis [PrEP], post-exposure prophylaxis [PEP], HIV testing services) in the South could further exacerbate HIV risk and related disparities. This, in turn, may undermine the EHE goals of reducing new HIV infections by 75% by 2025 and 95% by 2030 [1], potentially leading to higher transmission rates, increased economic burden, and worsened health outcomes.

In the scientific literature, little is known about the association between HIV risk and health care access among adults in the South. Utilizing the 2022 Behavioral Risk Factor Surveillance system (BRFSS), this study aimed to examine whether there was an association between HIV risk and healthcare access among U.S. adults in the South.

By understanding this association, study findings will inform public health strategies and policies aimed at improving healthcare access and reducing HIV-related disparities among populations at risk for HIV in the South. Additionally, findings will support the development of evidence-based public health programs to address socioeconomic and structural factors contributing to limited healthcare access in the South as part of efforts towards attaining the EHE goals.

## Materials and Methods

### Study Design

This study employed a cross-sectional study design utilizing data from the 2022 BRFSS, a complex sampling dataset by the CDC [18]. The purpose of the BRFSS is to collect uniform, state-specific data on health-related risk behaviors, chronic health conditions, health care access, and preventive service utilization, contributing to disease, death, and disability in the U.S [18].

### Data Source, Study Participants, and Sampling

The BRFSS is a nationally representative survey administered biennially by the Population Health Surveillance Branch of the CDC [18]. The BRFSS was first administered in 1984 across 15 states, and data on risky behaviors was collected monthly via telephone interviews [18]. In 2022 (January to December), BRFSS data was collected from adults who were 18 years or older, non-institutionalized, resided in the United States and participating territories - Guam, Puerto Rico, and the Virgin Islands, and had access to a landline or cellular telephone. However, this study only included responses from Southern states—Alabama, Arkansas, Delaware, Florida, Georgia, Kentucky, Louisiana, Maryland, Mississippi, North Carolina, Oklahoma, South Carolina, Tennessee, Texas, Virginia, West Virginia [19].

The BRFSS collects self-reported data from a randomly selected adult in a household via landline telephone surveys. However, for cellular telephone surveys, interviewers collect data from adults answering their phone who reside in a private residence or college housing. Based on the National Health Interview Survey preliminary results for July-December 2022, three-fourths (71.7%) of adults were wireless-only (cellular telephone) users, indicating that the percentage of households switching from landline telephones to cellular phones has significantly increased [18]. Therefore, including both landline and cellular telephones in a dual-frame survey improved the validity, data quality and representativeness of the BRFSS data.

State health departments conducted the field operations of BRFSS using a state-defined protocol with technical assistance from the CDC. Surveys were created jointly between state health departments, which conducted the interviews or relied on contractors to do so [18]. The data collected was then sent to the CDC where it was edited, processed, and then analyzed. The CDC then prepared state-specific data for the health departments of participating states for additional analysis/review, as well as a summary report. Thereafter, participating states utilized the BRFSS data for several purposes such as identifying disparities in the prevalence of leading U.S. public health issues, developing interventions, designing and evaluating public health policies and programs, and monitoring progress toward achieving state objectives, among others [18].

The BRFSS data is a two-stage cluster complex sampling dataset which requires that the data be weighted, clustered, and stratified using _LLWPWT (Final Weight), _PSU (Primary Sampling Unit), and _STRTR (Stratification) variables, respectively, before an analysis is performed. This process ensures the generalizability of results obtained from a sample to the entire population. In 2011, the BRFSS adopted a new weighting methodology called iterative proportional fitting or “raking” [18]. Raking incorporates data from cellular telephone surveys, enabling the introduction of additional demographic characteristics (education, marital status, home renter/owner) to age, race/ethnicity, and sex [18]. These additional characteristics improved the fidelity and generalizability of the BRFSS sample in accordance with demographic characteristics by individual states [18]. The 2022 BRFSS raking method included categories such as age by sex, detailed race and ethnicity groups, education levels, marital status, regions within states, sex by race and ethnicity, telephone source, renter or owner status, and age groups by race and ethnicity [18].

In BRFSS, a sample record is a single telephone number selected at random from a list of available numbers [18]. The landline samples for all 50 U.S. states and District of Columbia used a disproportionate stratified sample (DSS) design, while Guam, Puerto Rico and US Virgin Islands employed simple random-sample designs [18]. Prior to data collection, landline telephone sample records utilizing the DSS design were divided into two strata and sampled independently [18]. These strata included high-density and medium-density strata [18]. This assignment was based on the number of listed household numbers in its “hundred block”—a set of 100 telephone numbers that share an area code, prefix and first two digits (00–99) of a suffix along with all possible combinations for the last two digits [18]. The BRFSS samples both strata to obtain a probability sample of all households with telephones. To maintain confidentiality of responses provided by the respondent, specific variables (such as sub-state geographic identifiers, detailed race or ethnicity, and older than 80 years) in a given year are removed [18].

### Measures

Respondents were categorized according to socio-demographic variables of race/ethnicity (non-Hispanic (NH) White, NH Black, Hispanic, NH other, or NH multiracial), age in years (18–24, 25–34, 35–44, 45–54, 55–64, or 65 years or older); sex (male or female); sexual orientation (lesbian or gay, heterosexual, bisexual, or other); education level (less than high school, high school, or higher than school); income level (<$15k, $15k–<$ 35 K, $35k–<$50k, $50k–<$100,000k, $100k or more); employment status (employed for wages, self-employed, unemployed, retired); marital status (not married, married, divorced, widowed).

The outcome measure for this study is healthcare access, which comprises four distinct components: health insurance coverage, having a personal doctor, routine check-ups, and medical affordability, according to the CDC [20]. Each of these components was analyzed separately as a distinct dependent variable in a series of models. To assess health insurance coverage, participants were asked, “What is the current primary source of your health insurance?” Responses were dichotomized as “yes” (currently have at least one type of health insurance) or “no” (do not currently have health insurance). Regarding having a personal doctor, participants were asked, “Do you have one person or a group of doctors that you think of as your personal health care provider?” Responses were dichotomized as “yes” (had one or more personal doctors), or “no” (did not have a personal doctor). Regarding routine check-ups, participants were asked, “About how long has it been since you last visited a doctor for a routine checkup?” Responses were dichotomized as “Less than a year” or “One year or more.” Finally, to assess medical affordability, participants were asked the BRFSS item: “Was there a time in the past 12 months when you needed to see a doctor but could not because you could not afford it?” For consistency with other healthcare access variables, this item was recoded into a positively framed variable, where a “yes” response indicated medical affordability in the past year and a “no” response indicated a lack thereof. Table 1 presents an overview of all health care access variables.

The independent variable was HIV risk. Respondents were asked, “Do any of these situations apply to you: (1) you have injected any drug other than those prescribed for you in the past year, (2) you have been treated for a sexually transmitted disease or STD in the past year, (3) you have given or received money or drugs in exchange for sex in the past year, (4) you had anal sex without a condom in the past year, (5) you had four or more sex partners in the past year?” Responses included “yes,” “no,” “don’t know/not sure,” and “refused.” Data on HIV risk is collected bi-annually by the CDC, and at the time this manuscript was submitted, the 2022 BRFSS dataset represented the most up-to-date data available for the HIV risk variable.

Covariates controlled for included sociodemographic factors such as race/ethnicity, age group, sex, educational level, income level, employment status, and marital status.

All “don’t know/not sure” and “refused” responses were coded as “system missing” to prevent skewness of results, ensuring data integrity and accuracy. This approach aligns with methodologies used in prior studies employing BRFSS data [21, 22]. Additionally, a preliminary analysis of missing data indicated that less than 10% of the dataset was missing, and the data was determined to be missing at random. Consequently, it was disregarded, as such a low level of random missingness is unlikely to introduce bias into the analysis [23].

### Statistical Analyses

Descriptive statistics of sociodemographic, HIV risk, and healthcare access variables were performed using proc survey freq SAS procedures to obtain frequencies and proportions crucial to understanding the study population. This procedure generated weighted percentages for each of the variables.

Rao-Scott Chi-square tests were also performed using proc survey freq SAS procedures to examine weighted percentages of healthcare access by HIV risk, and statistically significant differences in health care access between U.S adults in the South at risk for HIV and those without risk. A level of statistical significance was established based on *p* < 0.05.

Weighted Logistic regression analyses were performed using proc survey logistic SAS procedures to examine the association between HIV risk and health care access, as well as to what extent an association exists, if any. Before conducting the logistic regression analysis, multicollinearity was evaluated and ruled out, as the independent variable and all covariates had variance inflation factor (VIF) values below 5. Simple logistic regression was performed for the unadjusted analysis while multiple logistic regression was performed for the adjusted analysis. The adjusted analysis accounted for sociodemographic covariates including race/ethnicity, gender, sexual orientation, education level, income level, employment status, and marital status to control for potential confounding factors that could influence the relationship between HIV risk and healthcare access. Odds ratios (OR) and adjusted odds ratios (aOR) obtained are essential outputs of logistic regression analyses as they serve as measures of association between predictor and outcome variables. OR and aOR were considered significant when the 95% confidence interval (95% CI) did not include 1.

All statistical analyses were performed using SAS version 9.4 [24].

**Table 1:** Health care access variables

Health Care Access	Questionnaire Item	Analytic Coding
Health Insurance Coverage	“What is the current primary source of your health insurance?”	Yes (currently have at least one type of health insurance) versus No (do not currently have health insurance)
Having a Personal Doctor	“Do you have one person or a group of doctors that you think of as your personal health care provider?”	Yes versus No
Routine Check-Up	“About how long has it been since you last visited a doctor for a routine checkup?”	< 1 year versus ≥ 1 year
Medical Affordability	“Was there a time in the past 12 months when you needed to see a doctor and could not because you could not afford it?”	Yes versus No (Variable recoded such that “Yes” indicates medical affordability and “No” indicates its absence).

## Results

### Descriptive Statistics of Socio-Demographic Characteristics, HIV Risk, and Health Care Access

The study sample comprised 121,966 respondents, representing 99,577,348 adults in the U.S. South. There were 55,027 males and 66,939 females in this study, making up 48% and 51% of our target population, respectively. The majority of our respondents were NH White (55%) followed by NH Black (18%), Hispanic (18%), NH Other race (5%), NH multiracial (3%) individuals. Majority of our respondents also identified as heterosexual (90%) followed bisexual (6%), other (2%), and lesbian or gay (2%).

Respondents’ ages were divided into six age groups: 18–24, 25–34, 35–44, 45–54, 55–64, and 65 or older, accounting for 12%, 17%, 17%, 16%, 16%, and 23% of our sample, respectively. Additionally, half of respondents were married (50%) and the majority had attained a degree higher than high school (59%). The largest group of respondents were also employed for wages (47%) and earned an annual income between $50,000 and $99,999 (29%).

Descriptive statistics revealed that 106,517 respondents answered the question on HIV risk. Of these, approximately 4,587 respondents, representing 5,361,890 (6%) adults in the South, reported that they were at risk of HIV.

Furthermore, most adults in the South had health insurance coverage (88%), a personal doctor (80%), could afford medical care in the past year (87%), and visited a doctor for a routine check-up in less than a year (77%). Table 2 provides detailed sociodemographic characteristics of the study population.

**Table 2:** Characteristics of the study population of U.S. Adults in the South, behavioral risk factor surveillance System, 2022

Characteristics	*n* ^a^	*N* ^b^	%^c^	[95% CI]
Age group				
18–24	6842	11,905,281	12.0	[11.5–12.4]
25–34	12,248	16,866,040	17.0	[16.5–17.4]
35–44	15,373	16,436,091	16.5	[16.1–16.9]
45–54	18,304	15,596,014	15.7	[15.3–16.1]
55–64	22,776	15,980,469	16.0	[15.7–16.4]
65 or older	46,423	22,793,453	22.9	[22.4–23.3]
Race/Ethnicity				
NH White	83,889	53,566,345	55.4	[54.9–56.0]
NH Black	18,796	17,342,290	18.0	[17.5–18.4]
NH Other Race	3780	5,172,039	5.4	[5.0–5.7]
NH Multiracial	2362	2,994,247	3.1	[2.9–3.3]
Hispanic	9461	17,529,358	18.1	[17.6–18.7]
Sex				
Male	55,027	48,181,839	48.4	[47.8–48.9]
Female	66,939	51,395,509	51.6	[51.1–52.2]
Sexual Orientation				
Lesbian or Gay	518	461,624	1.92	[1.6–2.2]
Heterosexual	31,121	21,682,393	90.0	[89.2–90.8]
Bisexual	1144	1,367,752	5.68	[5.0–6.3]
Other	618	579,918	2.41	[2.1–2.8]
Educational Level				
< High school	8820	12,558,169	12.7	[12.2–13.1]
High school	31,678	28,338,690	28.6	[28.1–29.1]
>High School	80,775	58,062,706	58.7	[58.1–59.2]
Income (USD)				
<$15k	6368	5,265,281	7.0	[6.7–7.3]
$15k–34,999k	22,972	18,697,304	24.9	[24.3–25.4]
$35k–49,999k	12,912	9,859,455	13.1	[12.7–13.5]
$50k–99,999	27,766	21,450,911	28.5	[28.0–29.1]
$100k or more	24,123	19,884,114	26.5	[25.9–27.0]
Employment Status				
Employed for wages	47,385	44,602,834	46.7	[44.7–48.7]
Self-Employed	9529	9,195,905	7.8	[6.8–9.0]
Unemployed	21,896	21,823,475	23.7	[21.9–25.4]
Retired	39,598	20,089,330	21.0	[20.6–21.4]
Marital Status				
Not married	27,326	31,104,843	31.6	[31.1–32.2]
Married	61,538	49,283,166	50.1	[49.5–50.7]
Divorced	16,258	10,567,355	10.7	[10.4–11.1
Widowed	15,495	7,414,457	7.5	[7.3–7.9]
HIV Risk				
Yes	4587	5,361,890	6.2	[6.0–6.5]
No	101,930	80,449,559	93.8	[93.5–94.0]
Health Insurance				
Yes	108,536	83,194,927	88.4	[88.0–88.8]
No	8090	10,910,587	11.6	[11.2–12.0]
Personal Doctor				
Yes	105,149	78,806,925	80.1	[79.6–80.6]
No	15,659	19,555,648	19.9	[19.4–20.4]
Routine Check-Up				
<1 year	98,819	75,066,255	77.3	[76.8–77.8]
≥1 year	20,809	22,062,785	22.7	[22.2–23.2]
Medical Affordability				
Yes	109,266	85,748,681	86.6	[86.2–87.0]
No	12,233	13,310,274	13.4	[13.0–13.8]

*n*^a^ unweighted number, *N*^b^ weighted number, *%*^c^ weighted percentage, *CI* confidence interval, *NH* non-Hispanic, USD United States Dollar

### Rao-Scott Chi-square Analysis

Results showed a statistically significant difference in healthcare access among U.S. adults in the South, revealing a lower weighted percentage in health insurance coverage (84% vs. 89%, p = < 0.001), having a personal doctor (69% vs. 82%, p = < 0.001), and visiting a doctor for a routine check in less than a year (67% vs. 78%, < 0.001) among individuals at risk of HIV compared to those not at risk. Additionally, a higher weighted percentage of being unable to afford medical care in the past year due to financial constraints (25% vs. 13%, p = < 0.001) was observed among individuals at risk for HIV compared to those without risk.

### Logistic Regression

Compared to those without HIV risk, results showed that U.S. adults in the South at risk for HIV had 1.55 higher odds of not having health insurance coverage (95% CI: 1.34–1.79), 2.00 higher odds of not having a personal doctor (95% CI: 1.78–2.25), 1.75 higher odds of delaying a routine doctor visit for one year or more (95% CI: 1.57–1.96), and 2.26 higher odds of not being able to afford medical care in the past year due to financial constraints (95% CI: 2.00–2.56) for unadjusted analysis.

After adjusting for covariates (race/ethnicity, age group, sex, educational level, income level, employment status, and marital status), results showed that U.S. adults in the South who were at risk for HIV had 1.69 higher odds of being unable to afford medical care in the past year due to financial constraints (95% CI: 1.45–1.97) compared to those not at risk. However, there was no association between HIV risk and health insurance coverage (aOR: 0.91; 95% CI: 0.76–1.08), HIV risk and having a personal doctor (aOR: 1.05; 95% CI: 0.91–1.22), and HIV risk and routine check-up (aOR: 0.98; 95% CI: 0.86–1.11).

Table 3 presents information on the simple and multiple logistic regression analyses performed.

**Table 3:** Logistic regression analysis on the association between HIV risk and healthcare access among U.S. Adults in the South, behavioral risk factor surveillance System, 2022

	Unadjusted Analysis			Adjusted Analysis		
HIV Risk	OR	[95% CI]	*p-value*	aOR	[95% CI]	*p-value*
Healthcare Access						
Health Insurance Coverage (Reference = Yes)						
Yes	**1.55**	**[1.34–1.79]**	**< 0.001**	0.91	[0.76–1.08]	0.276
No	1.00	Reference		1.00	Reference	
Having a Personal Doctor (Reference = Yes)						
Yes	**2.00**	**[1.78–2.25]**	**< 0.001**	1.05	[0.91–1.22]	0.480
No	1.00	Reference		1.00	Reference	
Routine Check-Up (Reference = Less than 1 Year)						
Yes	**1.75**	**[1.57–1.96]**	**< 0.001**	0.98	[0.86–1.11]	0.7054
No	1.00	Reference		1.00	Reference	
Medical Affordability (Reference = Yes)						
Yes	**2.26**	**[2.00–2.56]**	**< 0.001**	**1.69**	**[1.45–1.97]**	**< 0.001**
No	1.00	Reference		1.00	Reference	

*OR* Odds ratio, *aOR* Adjusted odds ratio, *CI* Confidence interval, Bolded—Significant finding

Covariates: race/ethnicity, gender, sexual orientation, education level, income level, employment status, and marital status

## Discussion

Using the 2022 BRFSS data, this novel study examined the association between HIV risk and health care access among adults in the US South, exploring whether there was a statistically significant difference in healthcare access between those at risk for HIV and those without risk. The results imply disparities in healthcare access among adults in the US South.

Findings suggest that adults at risk for HIV in the South have higher odds of accessing healthcare, including a higher likelihood of not having health insurance, a regular provider, or recent check-ups, as well as greater financial difficulty affording medical care. After adjusting for sociodemographic factors, the odds of being unable to afford medical care in the past year remained significantly higher (1.69 times) among individuals at risk for HIV in the South, highlighting persistent financial barriers to care. These findings highlight the economic disadvantage with which vulnerable populations at risk for HIV must contend, which may lead to underutilization of healthcare services such as HIV testing and PrEP prescriptions, subsequently causing poorer health outcomes [25].

Compared to other U.S. regions, the rate of HIV diagnoses remains highest in the South (52%) [3], and barriers to HIV preventive services among at-risk groups in the South may contribute to a lower likelihood of accessing healthcare. A mixed study by Wise et al. [26] which evaluated HIV testing uptake and barriers among 250 patients and 10 providers rtrttfacross three primary care clinics in Birmingham Alabama found that the most common barriers to HIV testing included cost and access to specialty care. A qualitative study by Arnold et al. [27] conducted in Jackson Mississippi to assess current barriers to PrEP use among young Black MSM found that structural barriers included cost of PrEP. According to McManus et al. [28], qualified health plans in the South disproportionately require prior authorization for PrEP, creating a major barrier to PrEP use.

Furthermore, the disruptions caused by the COVID-19 pandemic likely had a significant impact on both HIV risk and healthcare access. The pandemic restricted access to non-emergency clinical services such as PrEP delivery and sexual health counseling, as well as essential harm reduction programs including syringe exchange programs and HIV prevention and outreach programs [29–32], leaving individuals at risk for HIV, such as people who inject drugs or engage in transactional sex, with limited options for preventing HIV transmission. Additionally, the surge in mental health challenges, including anxiety, depression, and stress, contributed to higher rates of substance use, including injection drug use [32–34], which increases the risk of HIV transmission through shared needles. Moreover, substance use impairs judgment and decision-making, leading to riskier behaviors such as engaging in condomless sex or having multiple sexual partners [35–37]. Furthermore, the widespread implementation of shelter-in-place orders and social distancing measures caused economic hardships due to job losses and reduced incomes [32, 38], further straining medical affordability and making it more difficult for individuals to access necessary healthcare. This is particularly worrisome in regions like the South, where healthcare access and affordability challenges exist.

PrEP and HIV testing remain critical components of HIV prevention efforts in the United States and are largely accessible at no or low cost through the Affordable Care Act (ACA) and other initiatives [39, 40]. PrEP, a medication proven to significantly reduce the risk of HIV acquisition, received an “A” grade from the U.S. Preventive Services Task Force (USPSTF) in 2019, highlighting its substantial net benefit for individuals at increased HIV risk [41]. Similarly, routine HIV testing also holds an “A” grade from the USPSTF, affirming its importance for individuals aged 15–65, pregnant women, and those at increased risk [40]. This designation requires most insurance plans and Medicaid expansion programs to fully cover PrEP medication, clinic visits, and lab tests without cost-sharing, ensuring access even for individuals who have not met their annual deductible [39]. Additionally, the designation mandates most insurance plans and Medicaid expansion programs to provide free or low-cost HIV testing services, while traditional Medicaid programs are incentivized to offer such services [40].

Across Southern states, free or low-cost HIV testing initiatives further enhance accessibility to HIV preventive services. For example, the Tennessee Department of Health [42] offers free HIV lab testing across its 89 regional health departments, while the Louisiana state government facilitates free or low-cost testing through clinics and community programs located throughout the state [43]. Despite these efforts, barriers such as cost, limited access to HIV clinics, stigma, and privacy concerns persist. To address these barriers, the CDC launched the Together TakeMeHome (TTMH) project in 2023. This initiative prioritizes populations at higher risk for HIV, including racial and sexual minorities, by enabling discreet, at-home testing [44]. The program aims to distribute up to 1 million free HIV self-tests over five years, building on evidence that HIV self-tests are cost-effective [44]. These combined federal and state initiatives reflect a comprehensive national commitment to reducing financial barriers to HIV prevention.

Public health initiatives and policies on increasing healthcare access are crucial in addressing financial constraints faced by adults at risk for HIV in the South. Initiatives and policies recommended by Sullivan et al. [45] include enhancing reimbursement capacity for PrEP medications and services through policy reforms aimed at Medicaid expansion and maintaining access to Affordable Care Act compliant health plans, broadening STI screening initiatives, strengthening the integration of PrEP services with the delivery of positive STI results, and utilizing mHealth tools for periodic screening of high-risk groups for HIV, to improve access to healthcare services and lower out-of-pocket costs among populations at risk for HIV. Additionally, developing interventions and programs that mitigate HIV risk during pandemics, such as ensuring continued access to harm reduction services, expanding telehealth for sexual health counseling and PrEP delivery, and providing targeted financial support for at-risk populations, can help prevent the exacerbation of HIV-related disparities during public health emergencies. To achieve the goals of the EHE plan by the HHS, it is imperative that disparities in health care access affecting populations at risk for HIV are addressed, especially in the South.

### Strengths and Limitations

A strength of this study was the utilization of the 2022 BRFSS which employed a complex sampling design, enabling the generalizability of findings to the target population of U.S. adults in the South. Furthermore, the use of logistic regression analyses allowed researchers to observe differences in the results for both unadjusted and adjusted analysis thereby providing better insight on the association between HIV risk and healthcare access. However, this study is not without limitation. The BRFSS provides self-reported data which could introduce social desirability bias or recall bias when subjects intentionally misrepresent or misremember their HIV risk behaviors and healthcare accessibility [46, 47]. Additionally, the cross-sectional design of the data limits the ability to determine causality or establish temporal relationships between HIV risk and healthcare access.

Future research should utilize other study designs such as qualitative studies or longitudinal studies to further examine the relationship between HIV risk and healthcare access in the South, particularly medical affordability. Additionally, future studies should design and evaluate targeted interventions for increasing access to health care among U.S. adults at risk for HIV in the South.

## Conclusion

In 2022, there was a significant difference in healthcare access, particularly medical unaffordability, between U.S. adults in the South who were at risk for HIV compared to those who were not at risk. Findings confirm that there is association between HIV risk and healthcare access among U.S. adults in the South.

Given the disruptions caused by the COVID-19 pandemic and its impact on both HIV risk and healthcare access, it is essential for future studies to reexamine this association in the post-pandemic era. Such research will be critical in understanding the lasting effects of the pandemic and informing targeted interventions to address disparities in healthcare access and improve HIV prevention efforts.
